# Solitary lateral neck node metastasis in papillary thyroid carcinoma

**DOI:** 10.1186/1477-7819-12-109

**Published:** 2014-04-23

**Authors:** Seok-Mo Kim, Ki Won Chun, Ho Jin Chang, Bup-Woo Kim, Yong Sang Lee, Hang-Seok Chang, Cheong Soo Park

**Affiliations:** 1 Thyroid Cancer Center, Department of Surgery, Yonsei University College of Medicine, 211 Eonjuro, Gangnam-gu 135-720, Seoul, Korea

**Keywords:** thyroid, papillary, solitary, metastasis

## Abstract

**Background:**

Papillary thyroid carcinoma (PTC) is associated with a high incidence of regional node metastasis, but the patterns of lateral neck node metastasis (LNM) vary. Occasionally, a solitary LNM (SLNM) is seen in PTC patients. We therefore assessed whether selective single level node dissection is appropriate in PTC patients with SLNM.

**Methods:**

We retrospectively reviewed the medical records of 241 PTC patients who underwent total thyroidectomy with central neck dissection plus ipsilateral internal jugular node dissection (level II to IV) between January 2010 and December 2011. Of these patients, 51 had SLNM and 190 had multiple LNM (MLNM). The clinicopathologic characteristics of the two groups were compared.

**Results:**

Age, gender ratio, and numbers of lateral neck nodes harvested (29.4 ± 11.0 versus 30.3 ± 9.5; *P* = 0.574) were similar in the SLNM and MLNM groups. Mean primary tumor size was significantly smaller in the SLNM than in the MNLM group (1.03 cm versus 1.35 cm; *P* = 0.037). The proportion of patients with primary tumor ≤ 1 cm was significantly greater in the SLNM group (60.8% versus 38.4%; *P* = 0.006), whereas the proportion with maximal node size ≤ 0.7 cm (28.9% versus 73.3%; *P* <0.001) and the proportion with capsular invasion (62.7% versus 83.7%, *P* = 0.002) were significantly lower in the SLNM than in the MLNM group.

**Conclusions:**

Selective single level neck dissection can be considered as an alternative to systemic lateral neck dissection in PTC patients with SLNM, maximal metastatic node size ≤ 0.7 cm, and no extrathyroidal invasion.

## Background

Although regional lymph node metastasis is frequently observed in patients with papillary thyroid carcinoma (PTC), the patterns of lateral neck node metastasis (LNM) have been found to vary [1-5]. Some patients with PTC have solitary LNM (SLNM). LNM, whether solitary or multiple, has been associated with an increased risk of regional recurrence [1,4,6,7], and cervical lymph node recurrences have been reported in up to 31% of patients with PTC [8]. To reduce the risk of recurrence, various types of lateral neck dissection (LND) have been introduced in patients with clinically positive nodes [1,9,10]. Although many surgeons in other countries do not perform prophylactic LND, this method is preferred by Japanese surgeons [11]. Less invasive neck dissection in PTC patients may avoid perioperative morbidity and improve patient quality of life. Decisions regarding the extent of LND are usually based on predictable metastatic patterns. Less invasive neck dissection can be considered for patients with LNM confined to a single node. In the present study, we evaluated whether selective single level node dissection is appropriate in PTC patients with SLNM.

## Methods

Approval to retrospectively review the images and medical records of patients was obtained from the Institutional Review Board of Gangnam Severance Hospital, Yonsei University College of Medicine. In addition to approving the study protocol, the Institutional Review Board required neither patient approval nor informed consent for the review of records (#3-2012-0051).

Between 1 January 2010 and 31 December 2011, 656 PTC patients with LNM underwent surgery at the Thyroid Cancer Center, Gangnam Severance Hospital, Yonsei University College of Medicine, Korea. Patients were excluded if they had previously undergone thyroid surgery or radiotherapy. They were also excluded if they had other subtypes of PTC, other thyroid malignancies, bilateral thyroid cancer with bilateral neck node metastases, mediastinal metastasis, or other types of distant metastasis. Our study cohort consisted of 241 patients with conventional PTC who underwent total thyroidectomy with ipsilateral internal jugular node dissection (level II to IV). We did not include level V in patients with clinically negative level V nodes.

All patients underwent preoperative ultrasonography and/or neck computed tomography to evaluate the size and location of the tumor and the presence of cervical nodal metastases. Potential LNMs identified on preoperative imaging were further investigated by fine-needle aspiration biopsy, by measuring thyroglobulin concentrations in fine-needle aspirates, and/or by intraoperative frozen examination. Lateral neck nodes were classified into neck levels (II to IV) based on the criteria of the American Head and Neck Society [12].

Patients were divided according to the number of LNM, as determined from postoperative histopathologic records, into those with SLNM and those with multiple LNM (MLNM). The clinicopathological features of the two groups were compared, including patient sex and age; tumor size, location, multiplicity, bilaterality, and encapsulation; total number of retrieved lymph nodes; and maximal diameter of lateral neck nodes. Primary tumors were classified as being located in the upper, middle, and lower poles of the thyroid glands. When multiple foci were found, the dominant nodule was regarded as the primary carcinoma. Skip metastasis was defined as an LNM with no positive nodes in the central compartment [2]. Each node level was marked by the surgeon during LND.

Statistical analysis was performed using the Statistical Package for Social Science (SPSS) version 18.0 for Windows (SPSS, Inc, Chicago, IL, USA). Data in the two groups were compared using Student’s *t* test, the chi-square test, and Fisher’s exact test, as appropriate. Multiple logistic regression analysis was used to assess the statistical significance of the associations between SLNM and clinicopathologic factors. Odds ratios with 95% relative confidence intervals were calculated to determine the relevance of all potential predictors. A *P* value of < 0.05 was considered statistically significant.

## Results

The mean age of the 241 included patients was 43.9 ± 12.5 years (range 16 to 76 years), and the male to female ratio was 1:2.6 (67:174). The mean primary tumor size was 1.28 ± 0.97 cm. Multifocal and bilateral tumors were found in 84 (34.9%) and 44 (18.3%) patients, respectively. Most of the primary tumor lesions showed capsular invasion (79.3%). Thyroiditis was observed in 71 patients (29.5%), metastases to the central compartment in 173 patients (71.8%), and skip LNMs in 68 patients (28.2%). The mean maximal positive lymph node size by pathologic determination was 0.91 ± 0.62 cm (range 0.01 to 3.8 cm; Table 1).

**Table 1:** Patient demographics and clinical characteristics (n = 241)

**Characteristics**	**Results**
Age (mean and range)	43.9 ± 12.5 (16 to 76)
≥ 45 years	108 (44.8%)
< 45 years	133 (55.2%)
Sex (male/female)	67/174 (27.8%/72.2%)
Tumor size (cm, mean)	1.28 ± 0.97
> 1 cm	137 (56.8%)
≤ 1 cm	104 (43.2%)
Multifocality	
Yes/No	84 (34.9%)/157 (65.1%)
Bilaterality	
Yes/No	44 (18.3%)/197 (81.7%)
Capsular invasion	
Yes/No	191 (79.3%)/50 (20.7%)
Thyroiditis	
Yes/No	71 (29.5%)/170 (70.5%)
Central compartment metastases	173 (71.8%)
Skip metastases	68 (28.2%)
Solitary lymph node involvement	51 (21.2%)
Maximal lymph node size (cm, mean, and range)	0.91 ± 0.62 (0.01 to 3.8 cm)

**Figure 1:**
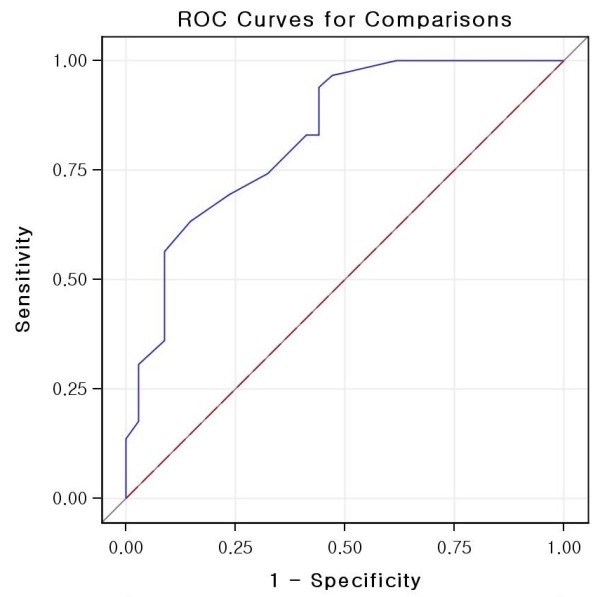
Receiver operating characteristic (ROC) curve for maximal metastatic node size and capsular invasion in the prediction of solitary lateral neck metastasis.

Of the 241 patients, 51 (21.2%) had SLNM and 190 (78.8%) had MLNM. Table 2 shows the demographic and pathologic characteristics of the two groups. Mean primary tumors were significantly smaller in patients with SLNM than in patients with MLNM (1.03 ± 0.59 cm versus 1.35 ± 1.05 cm; *P* = 0.037). Of the 51 patients with SLNM, 31 (60.8%) had primary tumors ≤ 1 cm in size. Capsular invasion was significantly less frequent (62.7% versus 83.7%; *P* = 0.002), whereas skip LNMs were significantly more frequently II (39.2% versus 25.3%; *P* = 0.049), in the SLNM than in the MLNM group. The mean maximal size of metastatic nodes was lower in the SLNM than in the MLNM group (0.40 ± 0.38 cm versus 1.03 ± 0.60 cm; *P* < 0.001). Using a cutoff of 0.7 cm, the metastatic node size had a positive predictive value of 40.2% and a negative predictive value of 91.7% for predicting the presence of SLNM. This maximal metastatic node size had a specificity of 76.5% and a sensitivity of 69.4%. A receiver operating characteristics curve relating maximal metastatic node size and SLNM is shown in Figure 1. There were no significant differences in age, sex, multifocality, or bilaterality between the SLNM and MLNM groups, and the numbers of lateral neck nodes harvested per patient were similar in the two groups. Logistic regression analysis revealed that capsular invasion of primary tumor and maximal metastatic node size > 0.7 cm were independent predictors of MLNM (Table 3). The distribution of SLNM according to primary tumor location is shown in Table 4. The highest incidence of SLNM was at level III (52.9%). None of the patients with primary tumors in the lower pole had SLNMs at level II.

**Table 2:** Comparisons of clinicopathologic variables between the solitary (SLNM) and multiple (MLNM) lateral compartment metastases

**Variables**	**Lateral compartment metastasis**		***P *** **value**
	**Group I (SLNM)**	**Group II (MLNM)**	
	**(n = 51)**	**(n = 190)**	
Age (years)	42.9 ± 12.0	44.2 ± 12.7	0.526
≥ 45	18 (35.3%)	90 (47.4%)	0.154
< 45	33 (64.7%)	100 (52.66%)	
Sex (male/female)	14 (27.5%)/37 (72.5%)	53 (27.9%)/137 (72.1%)	0.950
Tumor size (cm)	1.03 ± 0.59	1.35 ± 1.05	0.037
> 1 cm	20 (39.2%)	117 (61.6%)	0.006
≤ 1 cm	31 (60.8%)	73 (38.4%)	
Multifocality	211 (41.2%)	63 (33.2%)	0.322
Bilaterality	10 (19.6%)	34 (17.9%)	0.839
Thyroiditis	14 (27.5%)	57 (30.0%)	0.863
Capsular invasion	32 (62.7%)	159 (83.7%)	0.002
Harvested lateral neck node	29.4 ± 11.0	30.3 ± 9.5	0.574
Central compartment metastases	31 (60.8%)	142 (74.7%)	0.049
Skip metastasis	20 (39.2%)	48 (25.3%)	0.049
Maximal lymph node size (cm)	0.40 ± 0.38	1.03 ± 0.60	<0.001
> 0.7	12 (23.5%)	132 (69.5%)	< 0.001
≤ 0.7	39 (76.5%)	58 (30.5%)	

**Table 3:** Multivariate analysis of the association between multiple metastasis and clinicopathologic variables

**Variable**	**Odds ratio (95% confidence interval)**	***P*** **value**
Tumor variables		
Size (> 1 cm versus ≤ 1 cm)	1.810 (0.743 to 4.412)	0.192
Capsular invasion	1.952 (0.923 to 4.126)	0.039
Lymph node variables		
Central metastases	1.824 (0.739 to 4.506)	0.193
Maximal lymph node size	5.805 (2.540 to 13.270)	<0.001
(> 0.7 cm versus ≤ 0.7 cm)		

**Table 4:** Distribution of solitary lateral neck metastasis according to primary tumor location

**Neck level number**	**Upper n = 9 (%)**	**Mid n = 26 (%)**	**Lower n = 16 (%)**	**Total**
2	3 (33.3%)	2 (7.6%)	0 (0%)	5 (9.8%)
3	3 (33.3%)	12 (46.2%)	12 (75.0%)	27 (52.9%)
4	3 (33.3%)	12 (46.2%)	4 (25.0%)	19 (37.3%)

## Discussion

Cervical lymph node metastasis from PTC is common [1-5]. Although it remains unclear whether lymph node metastasis is associated with mortality, the risk of lymph node recurrence is thought to be increased when cervical lymph node metastasis is found at the time of diagnosis [1,9-11,13-15]. Re-operation for disease recurrence in the neck contributes to increased operative morbidity and medical costs [16-18]. Thus, surgeons should always perform a thorough preoperative evaluation for lymph node metastasis to determine the exact extent of neck dissection. The types of neck dissection in patients requiring LND include selective compartment, ipsilateral, and bilateral modified radical neck dissection [5,12,14,19]. Whether performed for diagnostic or therapeutic purposes, the role of LND is highly dependent on the metastatic pattern in lateral neck nodes. To our knowledge, this study is the first to provide information on the characteristics of primary PTCs in patients with SLNM. We hypothesized that the clinicopathologic features of the primary tumor may help predict the risk of SLNM and decrease the extent of LND, without increasing lateral neck recurrences.

Male gender, larger tumor size, T4 stage, and pathologic central lymph node metastasis have been found to increase the likelihood of LNM [1,3,4,20,21]. Our univariate and multivariate analyses showed that an absence of capsular invasion by the primary tumor and maximal node diameter ≤ 0.7 cm were significantly associated with SLNM. It is difficult to evaluate these factors by preoperative imaging methods, such as ultrasonography and computed tomography. However, intraoperative frozen sectioning of the primary tumor and metastatic lateral neck nodes can determine capsular invasion and lymph node size.

LNM is believed to occur initially in the central compartment and then spread to the lateral compartment [2,3,22-24]. Skip metastasis is thought to be a rare sub-phenomenon. Although skip metastasis occurs in only a small number of patients, it defeats the purpose of oncologic surgery and may result in disseminated distant metastases. We found that skip metastasis was more common in patients with SLNM than in those with MLNM, in agreement with our previous results [22].

Routes of lymphatic drainage in patients with PTC are both patient- and lesion-specific due to complicated lymphatic streams from the thyroid. The neck level at which metastasis is most frequent has been analyzed by subdividing tumor locations. For example, level II was the most frequent in patients with upper pole tumors, whereas levels III and IV were the most frequent in patients with lower and mid-pole tumors [16]. We found that 52.9% of patients with SLNM had metastases at level III. We observed no relationship between metastatic rates and neck level in patients with upper pole tumors, whereas metastases to level III nodes were more frequent in patients with lower and mid-pole tumors.

One limitation of this study was that we enrolled patients who underwent non-prophylactic but therapeutic LND. It is not possible to determine the true incidence of LNM in patients with PTC. The true incidence of LNM may affect the clinicopathologic features predicting SLNM. However, the decision to perform LND is usually based on clinical evidence for LNM. Although we evaluated clinically relevant nodal status that required initial LND, it is unclear whether prophylactic LND improves the prognosis of patients with PTC [19,25]. In contrast, clinically overt lymph node metastasis in the lateral compartment is a strong indicator of poor prognosis, and therapeutic LND has been recommended for patients with node metastases detected by ultrasonography [26-29]. Western guidelines therefore do not recommend prophylactic dissection of the lateral compartment [27].

Low-risk PTC had an excellent prognosis and a low mortality rate [6,13,15,17,29]. The treatment of it could be only thyroidectomy without node dissection [30]. Still, a proportion of patients with small tumors will experience recurrent or persistent disease. Careful risk stratification makes it possible to individualize treatment, avoiding unnecessary procedures, and guarantees a good long-term prognosis with a low recurrence risk. In this study, the SLNM group showed low-risk PTC characteristics in terms of small primary tumor size and no extrathyroidal extension.

## Conclusions

Our findings indicate that selective single level neck dissection can be considered an alternative to systemic LND in PTC patients with SLNM, maximal metastatic node size ≤ 0.7 cm, and no evidence of extrathyroidal invasion.

## Abbreviations

LND: lateral neck dissection; LNM: lateral neck node metastasis; MLNM: multiple lateral neck node metastis; PTC: papillary thyroid carcinoma; SLNM: solitary lateral neck node metastis.

## Competing interests

This study was financially supported by the faculty research grant of Yonsei University College of Medicine for 2011(6-2011-0066).

## Authors’ contributions

All authors read and approved the final manuscript.
